# CD4 T-cell expression of IFN-γ and IL-17 in pediatric malarial anemia

**DOI:** 10.1371/journal.pone.0175864

**Published:** 2017-04-20

**Authors:** Evans Raballah, Prakasha Kempaiah, Zachary Karim, George O. Orinda, Michael F. Otieno, Douglas J. Perkins, John Michael Ong’echa

**Affiliations:** 1 University of New Mexico Laboratories of Parasitic and Viral Diseases, Centre for Global Health Research, Kenya Medical Research Institute, Kisumu, Kenya; 2 Department of Medical Laboratory Sciences, Masinde Muliro University of Science and Technology, Kakamega, Kenya; 3 Department of Biochemistry and Biotechnology, Kenyatta University, Nairobi, Kenya; 4 Center for Global Health, Department of Internal Medicine, University of New Mexico, Health Sciences Centre, Albuquerque, NM, United States of America; 5 Department of Medical Laboratory Sciences, Kenyatta University, Nairobi, Kenya; Institut de recherche pour le developpement, FRANCE

## Abstract

In *Plasmodium falciparum* holoendemic transmission regions of western Kenya, life-threatening pediatric malaria manifests primarily as severe malarial anemia (SMA, Hb≤6.0 g/dL with any density parasitemia). To determine the role that CD4+ T-cell-driven inflammatory responses have in the pathogenesis of SMA, peripheral CD4+ T-cell populations and their intracellular production of pro-inflammatory cytokines (IFN-γ and IL-17) were characterized in children aged 12–36 months of age stratified into two groups: non-severe malarial anemia (non-SMA, Hb≥6.0 g/dL, *n* = 50) and SMA (*n* = 39). In addition, circulating IFN-γ and IL-17 were measured as part of a Cytokine 25-plex Antibody Bead Kit, Human (BioSource™ International). Children with SMA had higher overall proportions of circulating lymphocytes (*P* = 0.003) and elevated proportions of lymphocytes expressing IFN-γ (*P* = 0.014) and comparable IL-17 (*P* = 0.101). In addition, SMA was characterized by decreased memory-like T-cells (CD4+CD45RA-) expressing IL-17 (*P* = 0.009) and lower mean fluorescence intensity in memory-like CD4+ T-cells for both IFN-γ (*P* = 0.063) and IL-17 (*P* = 0.006). Circulating concentrations of IFN-γ were higher in children with SMA (*P* = 0.009), while IL-17 levels were comparable between the groups (*P* = 0.164). Furthermore, circulating levels of IFN-γ were negatively correlated with IL-17 levels in both groups of children (SMA: r = -0.610, *P* = 0.007; and non-SMA: r = -0.516, *P* = 0.001), while production of both cytokines by lymphocytes were positively correlated (SMA: r = 0.349, *P* = 0.037; and non-SMA: r = 0.475, *P* = 0.001). In addition, this correlation was only maintained by the memory-like CD4+ T cells (r = 0.365, *P* = 0.002) but not the naïve-like CD4+ T cells. However, circulating levels of IFN-γ were only associated with naïve-like CD4+ T cells producing IFN-γ (r = 0.547, *P* = 0.028), while circulating levels of IL-17 were not associated with any of the cell populations. Taken together, these results suggest that enhanced severity of malarial anemia is associated with higher overall levels of circulating lymphocytes, enhanced intracellular production of IFN-γ by peripheral lymphocytes and high circulating IFN-γ levels. In addition, the observed inverse relationship between the circulating levels of IFN-γ and IL-17 together with the reduction in the levels of memory-like CD4+ T cells expressing IL-17 in children with SMA may suggest possible relocation of these cells in the deeper tissues for their pathological effect.

## Introduction

Malaria continues to be a major public health problem, which resulted in about 214 million cases and over 438,000 deaths world-wide in 2015 [1]. The vast majority of cases (~88%) and deaths (>90%) occur in sub-Saharan Africa, largely in immune-naive children under five years of age [1]. *Plasmodium falciparum* is responsible for over 98% of the morbidity and mortality borne by African children [2]. The two primary severe disease outcomes of malaria are cerebral malaria and severe malarial anemia (SMA) with the distribution of these severe forms being largely dependent on malaria transmission intensity [3]. Although cerebral malaria is more common in older children in regions of low-to-moderate transmission intensity, SMA is the primary manifestation seen in children with median ages of 15 months (IQR 9–25 months) that live in holoendemic transmission areas [4]. As such, in the current study area in western Kenya, severe malaria primarily manifests as SMA (hemoglobin (Hb) < 6.0g/dL [5] peaking in children of 7–24 months of age [6].

The pathogenesis of pediatric malarial anemia (MA) in holoendemic *P*. *falciparum* transmission areas is largely determined by the degree of red blood cell (RBC) destruction and production [7–9]. Chronic forms of *P*. *falciparum* resulting from persistent parasitemia and repeated infections are a primary cause of enhanced anemia severity in African children [5, 6, 10–13] with anemia often persisting even after successful clearance of parasitemia [14] due to bone-marrow suppression [15] which is characterized by dyserythropoiesis and infective erythropoiesis [13, 16, 17]. Suppressed and ineffective erythropoietic responses are associated with enhanced production of macrophage-derived inflammatory cytokines [18, 19] as a consequence of prolonged immune activation [20] driven, at least in part, through interactions with CD4+ T-cells [21, 22]. For example, production of IL-12 and IL-23 by macrophages/monocytes and other myeloid antigen presenting cells (APCs) induce naïve and memory CD4+ T-cells to produce IFN-γ and IL-17, respectively [21, 22]. Although IFN-γ may induce protective immune responses against parasitemia, re-infections, anemia and clinical malaria [23–26], increased IFN-γ production has also been associated with enhanced pathogenesis [27–29]. Enhanced pathogenesis, and particularly anemia, in the context of elevated IFN-γ is consistent with murine studies showing that treatment with CpG-ODN [30] and acute toxoplasmosis [31] cause increased IFN-γ levels that appear to contribute to suppression of erythropoiesis. The myriad of effects that IFN-γ has on pathophysiological processes in bone marrow was recently reviewed by de Bruin *et al*. [32].

IL-17 is a pro-inflammatory cytokine that has been implicated in the inflammation of autoimmune diseases in response to IL-23 production [33]. We have also shown that differing levels of IL-12 and IL-23 are associated with disease severity in children with malarial anemia [34–36]. However, the effect of altered IL-12 and IL-23 on conditioning naïve and memory CD4+ T-cells to produce IFN-γ and IL-17, respectively, during an acute malarial infection is unknown. Recent studies in murine models reported that chronic malaria infection was associated with an increased proportion of effector memory CD4+ T-cells that produced IFN-γ and TNF-α which were effective at delaying and reducing parasitemia and pathology [37]. Additional studies in murine models reported involvement of IFN-γ and the IL-17/IL-23 axis in malaria associated pathology [38]. However, human studies from Cameroon reported that enhanced production of IFN-γ and IL-17 were associated with protection against pregnancy-associated malaria [39]. Recent studies in chronic inflammatory diseases reported plasticity in the ability of effector Th17 cells to produce IL-17 and/or IFN-γ following conditioning with IL-23 [40, 41]. Similarly, Perez-Mazliah and Langhorne [42] recently reviewed plasticity in CD4 T-cell sub-sets during malaria infections.

Based on previous work from our laboratory and others [34–36, 43], we investigated the hypothesis that enhanced CD4+ T-cell-derived IFN-γ and/or IL-17 promoted enhanced pathogenesis of malarial anemia. As such, we characterized CD4+ T-cell populations and their intracellular production of IFN-γ and IL-17 in children with SMA and non-SMA. Since malarial anemia severity is significantly influenced by co-infections, such as human immunodeficiency virus type 1 (HIV-1) [44], bacteremia [45, 46] and helminthic infections [47], all children with these infections were excluded from the analyses. We show that children presenting with SMA have elevated levels of lymphocytes producing IFN-γ compared to non-SMA children. In addition, we demonstrate that children with SMA had decreased levels of memory-like CD4+ T-cells expressing IL-17 and elevated circulating IFN-γ levels. Taken together, these results implicate both IFN-γ and IL-17 in the pathogenesis of SMA.

## Materials and methods

### Ethics statement

The study was approved by the National Ethical Review Committee of the Kenya Medical Research Institute and the Institutional Review Board of the University of New Mexico (SSC696/SSC1377). All the parents or legal guardians of the children gave written informed consent in their language of choice (Dholuo, Kiswahili or English) before enrollment into the study. The study was conducted in accordance with the Declaration of Helsinki and Good Clinical Practice.

### Study area

The current study was performed at Siaya County Referral Hospital (SCRH) in Siaya County, western Kenya. SCRH is situated in a *P*. *falciparum* holoendemic transmission area that reported increased pediatric malarial admissions despite recent anti-malarial interventions [48]. SMA is the primary clinical manifestation of severe malaria in children under the age of 5 years, peaking in children aged 7–24 months [5, 6]. Previous studies observed that 53% of all the malaria-related deaths in hospitalized children under the age of 3 years were due to SMA [49]. The manifestation of pediatric MA in the study population has been described in detail elsewhere [50].

### Study population

Children (*n* = 89, aged 12–35 months) presenting at the SCRH were recruited into the study. Children with *P*. *falciparum* parasitemia (any density) were stratified based on Hb levels as follows: non-SMA (Hb>6.0g/dL; *n* = 50) and SMA (Hb≤6.0 g/dL; *n* = 39). SMA was defined with an Hb cutoff of 6.0 g/dL based on previous investigations of >14,000 longitudinal Hb distributions in children aged less than 48 months in western Kenya [5]. Since HIV-1 promotes anemia in children with falciparum malaria [44], only HIV-1 negative children were included in analyses of the present study. HIV-1 status was determined by two rapid serological antibody tests and HIV-1 proviral DNA PCR tests as previously described [44]. Similarly, bacteremic children, as well as those with hook-worm infections, were excluded from the study. All the study participants were also free from cerebral malaria, (which is a rare occurrence in this high malaria transmission area [6]), non-falciparum malaria parasites, and hypoglycemia. Children with malaria were treated according to the Ministry of Health, Kenya (MOH) guidelines using Coartem® (artemether and lumefantrine) for uncomplicated malaria and intravenous quinine for severe malaria. Supportive care and blood transfusions were administered according to MOH guidelines. All blood samples were obtained prior to antimalarial and/or any other treatment interventions.

### Parasitemia and Hb levels determination

Venous blood (<3.0mL) samples were obtained from the study participants after the parent/guardian signed an informed consent. Thick and thin peripheral blood smears were prepared from venous blood samples and stained with Giemsa reagent for malaria parasite identification and quantification by microscopy. Asexual malaria parasites were counted against 300 leukocytes, and parasite densities were determined by multiplying the parasite count by the total leukocyte counts from an automated hematology analyzer (Beckman Coulter^®^ AcT diff2™, Beckman-Coulter Corporation, Miami, USA). Hb levels were determined using the automated hematology analyzer (Beckman Coulter^®^ AcT diff2™, Beckman-Coulter Corporation, Miami, USA).

### Characterization of CD4+ T cell populations

**Fig 1:**
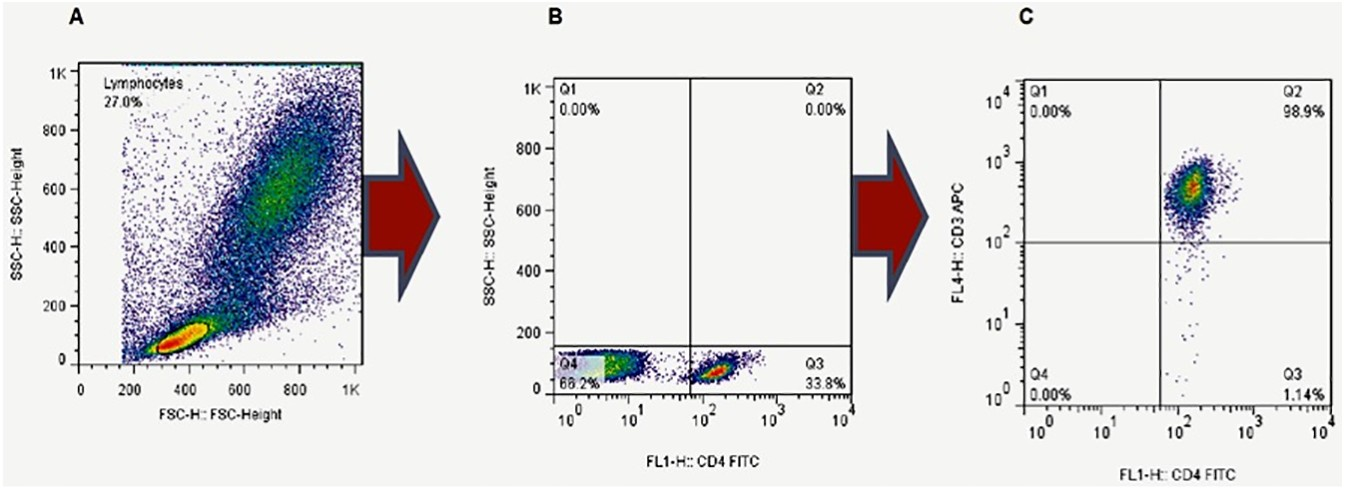
Characterization of CD4+ T-cell sub-populations. **(A)** Initial gating was performed on lymphocytes in the overall cell population during acquisition. **(B)** The CD4+ T-cell population was then selected by gating on CD4+ cells in the lymphocyte cell population during analyses. **(C)** Due to the limitation of the four-color cytometer, the populations of CD4+ cells used in subsequent analyses were CD4+ cells from the lymphocyte gate. To determine the proportion of CD4+ cells that were T-cells (CD3+CD4+), expression of CD3 by the CD4+ cells (gated in B) was determined. The median proportion of CD3+CD4+ (T-cells) among the CD4+ cells was 98.9%, suggesting that the CD4+ gating selected an enriched population of CD4+ T-cells.

Approximately 500 μL of venous blood was obtained and stained with mouse anti-human (APC CD3, BD Pharmingen^TM^ Cat.555335; FITC CD4, BD Pharmingen^TM^ Cat.555346; and PE-Cy™^5^ CD45RA, BD Pharmingen^TM^ Cat.555490) antibodies. Briefly, venous blood was diluted with an equal volume of plain RPMI-1640 media and aliquots of 100μL were pipetted into snap-cap tubes. The blood-media mixture was then stained with 10 μL of fluorochromed surface antibodies and incubated at 4°C for 30 minutes. The red blood cells (RBCs) were then lysed using cold 1X BD Pharm Lyse^TM^ lysing buffer (Cat.555899) at room temperature for 10 minutes. The cells were washed twice in cold wash buffer (5g Bovine serum albumin (BSA) + 0.5g Sodium azide in 500mL Phosphate buffered saline (PBS)) and fixed in 1% paraformaldehyde at 4°C. Cells were then acquired using a four-color FACSCalibur within 24 hours of staining. Lymphocytes were acquired and stored on gate R1 (Fig 1A). These were further gated as CD4+ and CD4- lymphocytes as shown in Fig 1B. In order to determine the proportion of CD4+ lymphocytes that were T cells, a third gate for CD3+ was used (Fig 1C). This gate demonstrated that ~98.0% (median value) of the CD4+ lymphocytes were (CD3+) T cells.

### Intracellular cytokine stimulation assays

Since all children in the study had malaria parasitemia, measurement of *ex vivo* responses to malaria infections was determined by obtaining ~500 μL of venous blood that was diluted with an equal volume of plain RPMI 1640 media followed by stimulation with 0.1μg/mL anti-CD28/CD49d (Cat.347690) in the presence of monensin (0.5 μg/mL) for IL-17, or Brefeldin A (0.5 μg/mL) for IFN-γ and incubated at 37°C and 5% CO_2_ for six hours. After six hours of incubation cells were fixed in 1% paraformaldehyde at 4°C, permeabilized with 0.1% saponin (0.1g saponin in 100mL PBS) for thirty minutes and then stained with 10 μL of surface antibodies (CD4 and CD45RA), and intracellular cytokine antibodies (FITC IFN-γ, BD Pharmingen^TM^ (Cat.554551) and PE IL-17, R&D Systems, (Cat.ICP3171P)) and incubated at 4°C for thirty minutes. The cells were then processed following the steps described above and acquired using a four-color FACSCalibur immediately after staining.

### Determination of circulating cytokine levels

Plasma samples were obtained from venous blood and snap-frozen at -80°C until use. Concentrations of IL-17 and IFN-γ were determined using the human Cytokine 25-plex Ab Bead Kit, (BioSource™ International) according to the manufacturer’s protocol. Plates were read on a Luminex 100™ system (Luminex Corporation) and analyzed using the Bio-Plex Manager Software (Bio-Rad Laboratories), with detection limits of 4.0 pg/mL (IFN-γ) and 10.0 pg/mL (IL-17).

### Data analysis

All CD4+ frequencies presented are data that resulted after compensation using unstained, single-stained and multiple-stained cells on FlowJo 7.6.3 software (TreeStar, Ashland, OR, USA). Proportions of CD4+ T-cells expressing the various surface markers and the intracellular cytokines were analyzed using FlowJo. MFIs were calculated by the geomean of the population of interest in FlowJo. Statistical analyses were performed using SPSS^®^ software (version 19.0, IBM SPSS Inc., IL, USA). Between group comparisons were performed by Mann-Whitney U tests. Proportional differences between the clinical groups was determined using χ^2^-tests. The relationships between circulating cytokine levels and intracellular cytokine expression by different CD4+ T cell sub-populations were determined by Spearman’s correlation coefficient. Statistical significance was set at *P*≤0.050.

## Results

### Characteristics of the study participants

The demographic and clinical characteristics of the study participants are summarized in Table 1. Children in the two clinical categories (non-SMA and SMA) were comparable in gender (*P* = 0.673), axillary temperature (*P* = 0.422), parasite density (*P* = 0.256), prevalence of high-density parasitemia (HDP, ≥10,000 parasites/μL) (*P* = 0.259) and carriage of sickle-cell trait (*P* = 0.257). However, children presenting with SMA were significantly younger than those with non-SMA (*P* = 0.019). In addition, children with SMA had significantly low levels of reticulocyte production index (RPI; *P* = 0.007), an indicator for erythropoietic response based on reticulocyte counts correcting for the degree of anemia [51]. Furthermore, children with SMA had elevated levels of peripheral white blood cell (WBC) counts (*P* = 0.014), proportion of circulating lymphocytes (*P* = 0.003) and reduced Hb (*P* = 0.001) relative to the non-SMA group.

**Table 1:** Demographic, clinical and laboratory characteristics of study participants.

Characteristic	Non-SMA	SMA	*P*
Participants, *n*	50	39	
Gender, female, *n* (%)	25 (50.00)	17 (43.59)	0.673^*a*^
Age, months	23.00 (13)	18.00 (14)	**0.019**^***b***^
Axillary temperature, °C	38.00 (1.4)	38.00 (1.2)	0.422^*b*^
Hemoglobin, g/dL	9.60 (2.5)	5.00 (1.6)	**<0.001**^***b***^
Reticulocyte production index	0.52 (0.34)	0.33 (0.41)	**0.007**^***b***^
White blood cells, (×10^9^/L)	10.60 (5)	13.30 (8)	**0.014**^***b***^
Lymphocytes (%)	33.65 (24)	40.20 (13)	**0.003**^***b***^
Parasite density, /μL	31,200 (131,761)	25,604 (91,327)	0.256^*b*^
High-density parasitemia, *n* (%)	37 (74.00)	23 (58.97)	0.259^*a*^
Sickle-cell trait, *n* (%)	10 (20.00)	4 (10.26)	0.257^*a*^

Data are presented as medians (interquartile range; IQR) unless otherwise noted. Children (*n* = 89) were categorized into non-SMA (*n* = 50, Hb≥6.0 g/dL with any density parasitemia) and SMA (*n* = 39, Hb<6.0 g/dL with any density parasitemia). Statistical significance was set at *P*<0.05.

^*a*^Statistical significance was determined by Chi-square analysis.

^*b*^Statistical significance was determined by Mann-Whitney U tests.

### Characterization of distinct CD4+ T-cell populations

The gating strategy used in characterizing the CD4+ T-cell populations is outlined in Fig 1 **(and S1–S3 Figs)**. The lymphocyte population was gated on to provide the overall cell populations during acquisition (Fig 1A). This gate was then dissected into CD4+ and CD4- populations (Fig 1B). The CD4+ T-cell populations reported were based on the CD4+ cells selected by this gating. However, to confirm the proportion of CD4+ cells that were T-cells (CD3+CD4+), cells expressing CD3 among the CD4+ cells (Fig 1B) were also determined. We observed that the CD4+ cells isolated (Fig 1B**)** were mainly CD3+ T-cells (Median (IQR) ~98.0% (3.15), Fig 1C).

### Lymphocytes expressing intracellular IFN-γ and IL-17 are elevated in children with SMA

**Fig 2:**
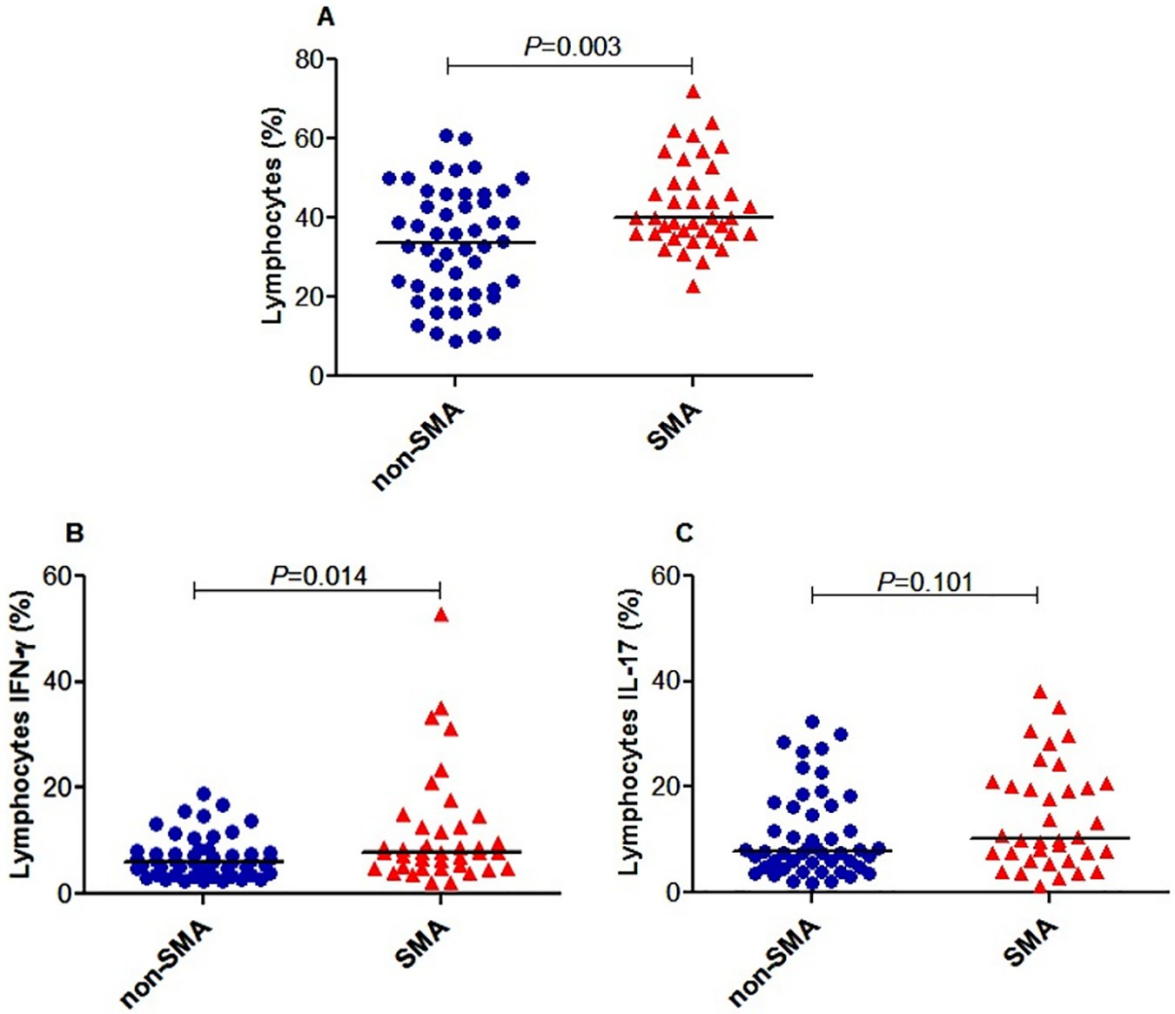
Lymphocytes expressing IFN-γ and IL-17 in acute malaria. Data are presented as proportions (%). The proportions were determined by flow cytometry immediately after staining. The proportions of different lymphocyte populations were determined using FlowJo Software (TreeStar, Ashland, OR, USA). The line in the middle represents the median value. Between groups comparisons were performed by Mann-Whitney U test. **(A)** The proportion of lymphocytes was higher in the SMA group relative to non-SMA group. **(B)** The proportion of lymphocytes expressing IFN-γ was higher in the SMA group relative to non-SMA group. **(C)** The proportion of lymphocytes expressing IL-17 was higher in the SMA group relative to non-SMA group.

Preceding the examination of the role of CD4+ T-cells in malarial immunity, the distribution of lymphocytes and their intracellular cytokines were examined. Children with SMA had elevated lymphocytes (median (IQR) 40.00% (13.00)) relative to the non-SMA group (33.65% (25.00); *P =* 0.003; Fig 2A). Further analyses demonstrated that the SMA group had significantly higher circulating lymphocytes expressing IFN-γ (7.78% (7.71)) and comparable IL-17 (10.30% (14.25)) relative to children with non-SMA (6.03% (4.45) and 7.80% (11.55); *P =* 0.014 and *P =* 0.101, respectively; Fig 2B and 2C).

### Memory-like CD4+ T-cells expressing IL-17 are decreased in children with SMA

**Fig 3:**
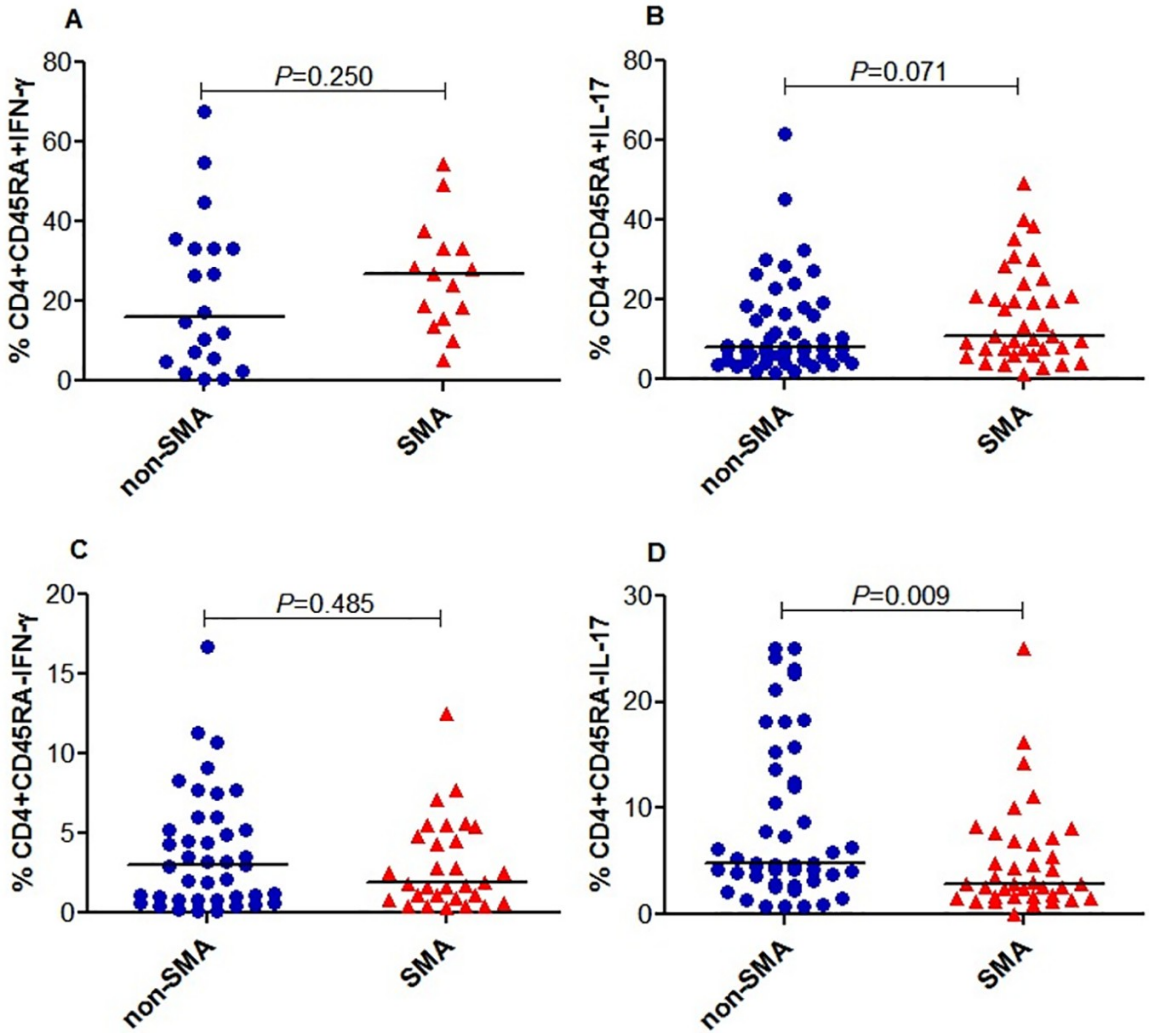
Memory-like CD4+ T-cells expressing IFN-γ and IL-17 in acute malaria. Data are presented as proportions (%). The proportions were determined by flow cytometry immediately after staining. The proportions of different lymphocyte populations were determined using Flowjo Software (TreeStar, Ashland, OR, USA). The line in the middle represents the median value. Between groups comparisons were performed by Mann-Whitney U test. The proportion of CD4+ T-cells was comparable between the groups (data not shown). **(A)** The proportion of naïve-like CD4+ T-cells expressing IFN-γ was comparable between the groups. **(B)** The proportion of naïve-like CD4+ T-cells expressing IL-17 was comparable between the groups. **(C)** The proportion of memory-like CD4+ T-cells expressing IFN-γ was comparable between the groups. **(D)** The proportion of memory-like CD4+ T-cells expressing IL-17 was decreased in SMA relative to non-SMA group.

Prior to characterizing naïve-like (CD4+CD45RA+) and memory-like (CD4+CD45RA-) CD4+ T-cells expressing IFN-γ and IL-17, we compared the distribution of CD4+ T-cell counts between the groups. These results demonstrated that the proportion of CD4+ T-cells was comparable between SMA and non-SMA groups (median (IQR) SMA 52.73% (11.51) and non-SMA 55.60% (16.17); *P =* 0.394; Data not shown). In addition, naïve-like CD4+ T-cells expressing IFN-γ were non-significantly elevated in the SMA group (median (IQR) SMA 26.90% (17.50) and non-SMA 16.00% (28.28); *P =* 0.250; Fig 3A). Naïve-like CD4+ T-cells expressing IL-17 were also non-significantly higher in the SMA group (median (IQR) SMA 10.80% (16.70) and non-SMA 8.00% (12.12); *P =* 0.071; Fig 3B). Further analysis demonstrated that the memory-like CD4+ T-cells expressing IFN-γ were non-significantly reduced in the SMA group (median (IQR) SMA 1.90% (4.06) and non-SMA 3.03% (4.79); *P =* 0.485; Fig 3C). Memory-like CD4+ T-cells expressing IL-17 were significantly lower in children with SMA (median (IQR) SMA 2.86% (5.37) and non-SMA 4.85% (12.20); *P =* 0.009; Fig 3D).

### Intensity of IFN-γ and IL-17 production by memory-like CD4+ T-cells is decreased in children with SMA

**Fig 4:**
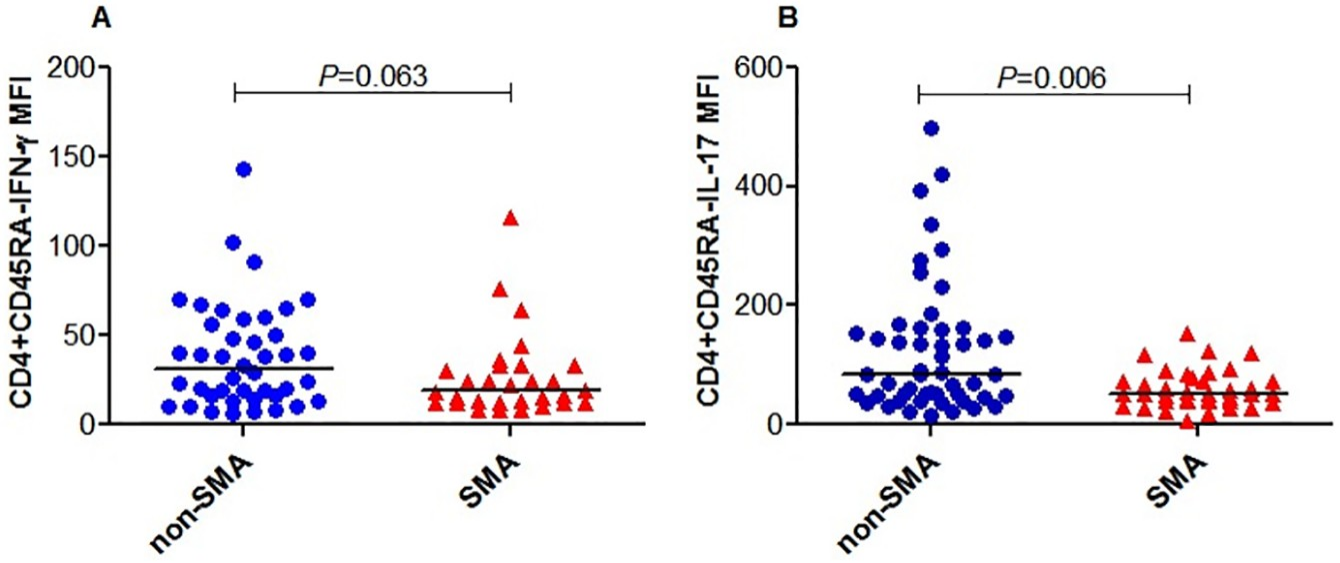
Intensity of IFN-γ and IL-17 production by memory-like CD4+ T-cells. Data are presented as mean fluorescence intensity (MFI). The MFI was determined by FlowJo Software (TreeStar, Ashland, OR, USA). The line in the middle represents the median value. Between groups comparisons in the clinical categories were performed by Mann-Whitney U tests. **(A)** MFI of IFN-γ in CD4+CD45RA- cells. **(B)** MFI of IL-17 in CD4+CD45RA- cells.

The intensity of T-cell response is more informative than the proportions of the cells producing the response [52], therefore we extended the above investigations by examining the intensity of IFN-γ and IL-17 in memory-like T helper cells. The strength of expression of IFN-γ and IL-17 by memory-like CD4+ T-cells (CD4+CD45RA-) was determined by calculating the mean fluorescence intensity (MFI). These results demonstrated that the MFI for IFN-γ in memory-like CD4+ T-cells (CD4+CD45RA-IFN-γ) was reduced in children with SMA (median (IQR) SMA, 18.83 (20.74) and non-SMA, 31.17 (41.93); *P =* 0.063; Fig 4A). The MFI for IL-17 in memory-like CD4+ T-cells (CD4+CD45RA-IL-17) was also significantly lower in the SMA group (median (IQR) SMA, 51.52 (35.61) and non-SMA, 83.41 (111.58); *P =* 0.006; Fig 4B).

### Relationship between circulating cytokines and SMA

**Fig 5:**
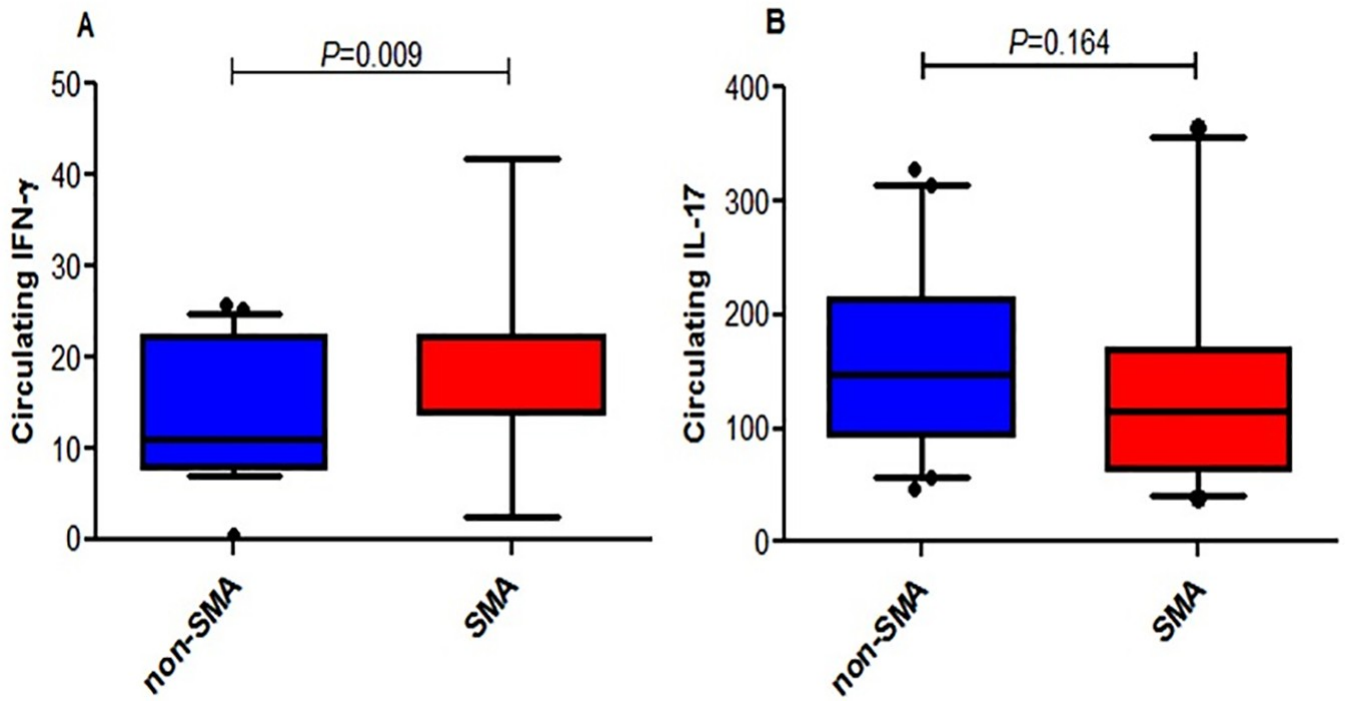
Circulating levels of IFN-γ and IL-17 in children with differing malarial anemia severity. Box-plots depict the data where the box represents the interquartile range, the line through the box is the median, and whiskers illustrate the 10th and 90th percentiles. Circulating IFN-γ (*n* = 64; non-SMA = 42; SMA = 22) and IL-17 (*n* = 64; non-SMA = 42; SMA = 22) concentrations were determined in plasma using the human Cytokine 25-plex Ab Bead Kit, (BioSource™ International) according to the manufacturer’s protocol. Plates were read on a Luminex 100™ system (Luminex Corporation) and analyzed using the Bio-Plex Manager Software (Bio-Rad Laboratories). Between groups comparisons in the clinical categories were performed by Mann-Whitney U tests. **(A)** Circulating IFN-γ levels between non-SMA and SMA. **(B)** Circulating IL-17 levels between non-SMA and SMA.

To determine the overall production of IFN-γ and IL-17, circulating levels of the cytokines were measured in the two groups of children. Children with SMA had elevated circulating levels of IFN-γ relative to the non-SMA group (median (IQR) SMA 22.22 (8.41) and non-SMA 11.04 (14.34); *P* = 0.009; Fig 5A). Circulating IL-17 levels were comparable between the two groups (median (IQR) SMA 115.00 (105.84) and non-SMA 147.00 (120.76); *P* = 0.164; Fig 5B).

### Relationship between circulating cytokine levels and intracellular cytokine expression by different CD4+ T cell sub-populations

Overall, circulating levels of IFN-γ and IL-17 were negatively correlated with each other (r = -0.464, *P* = 0.0001). This pattern was further observed independently in the SMA (r = -0.610, *P* = 0.007) and non-SMA (r = -0.516, *P* = 0.001) groups. However, production of both cytokines by lymphocytes was positively correlated (r = 0.453, *P* = 0.0001) and independently in the SMA (r = 0.349, *P* = 0.037) and non-SMA (r = 0.475, *P* = 0.001) groups. Furthermore, there was no relationship between the production of both cytokines by naïve-like CD4+ T cells (r = 0.232, *P* = 0.425) while there was a positive correlation in the production of both cytokines by memory-like CD4+ T cells (r = 0.365, *P* = 0.002). In addition, the circulating levels of IFN-γ were positively correlated with intracellular expression of IFN-γ by naïve-like CD4+ T cells (r = 0.547, *P* = 0.028), but not memory-like CD4+ T cells (r = -0.214, *P* = 0.179) nor lymphocytes (r = 0.144, *P* = 0.298). There was no relationship between the circulating levels of IL-17 with intracellular expression of IL-17 by naïve-like CD4+ T cells (r = 0.252, *P* = 0.330), memory-like CD4+ T cells (r = 0.096, *P* = 0.492), nor lymphocytes (r = 0.099, *P* = 0.483). Similarly, there was no relationship between the circulating levels of the two cytokines individually and their expression by the different cell sub-populations neither in the SMA nor the non-SMA groups (*P*>0.05 for all comparisons).

## Discussion

Understanding the mechanisms through which malaria-induced inflammatory responses modulate SMA pathogenesis will aid in the rational design of therapeutic interventions. A number of novel findings emerged in the current studies including data that suggest that naïve-like CD4+ T-cells (CD4+CD45RA+) are elevated in children with SMA relative to children with non-SMA. In addition, children with SMA had higher circulating lymphocytes expressing IFN-γ. Moreover, as previously reported by our group, children with SMA had higher concentrations of circulating IFN-γ relative to the non-SMA group [53]. In addition, we observed that circulating levels of IFN-γ were negatively correlated with circulating levels of IL-17 in both groups of children, while expression of both cytokines by lymphocytes was positively correlated. Further analysis demonstrated that circulating levels of IFN-γ were positively associated with naïve-like CD4+ T cells expressing IFN-γ while circulating levels of IL-17 were not associated with intracellular expression of IL-17 by any of the cell populations. Taken together, these results suggest that high levels of lymphocyte-derived IFN-γ may contribute to the pathogenesis of pediatric SMA. Alternatively, the observed low levels of circulating memory-like CD4+ T-cells producing IFN-γ and IL-17 in children with SMA may also suggest possible sequestration in the deeper tissues where they could induce pathogenic processes.

We hypothesized that since pediatric SMA is driven by a sustained inflammatory response following chronic plasmodial infection [5, 10–13, 54], specific populations of immune cells should contribute to the production of pro-inflammatory cytokines (e.g., IFN-γ and IL-17). In addition, recent studies by Boyle *et al*. [55] illustrated that there are malaria-specific CD4+ T-cells that produce differing cytokine responses following stimulation with *P*. *falciparum*-infected red blood cells (iRBCs). Therefore to test this hypothesis, we characterized the *ex vivo* ability of peripheral lymphocytes and CD4+ T-cell sub-populations to produce IFN-γ and IL-17 in acutely infected children presenting with non-SMA and SMA. In agreement with previous reports, children presenting with SMA had leukocytosis [56, 57]. In addition, our observation of elevated levels of lymphocytes expressing IFN-γ in children with SMA and memory-like CD4+ T-cells expressing IL-17 in children with non-SMA, suggests *in vivo* induction of these cells as has been reported in animal models [43, 58–60]. Furthermore, our observation that children with SMA had lymphocytosis, high IFN-γ expression by peripheral lymphocytes and the observed correlation between circulating levels of IFN-γ and naïve-like CD4+ T cells expressing IFN-γ may implicate naïve CD4+ T cells in the production of high levels of circulating IFN-γ in children with SMA. Although recent studies have been able to characterize cytokine profiles of pediatric CD4+ T-cell populations [55, 61, 62], these have related the cytokine responses with malaria protection. However, studies among Malian children investigated the cytokine profiles during the control of malaria-induced inflammation and observed a more enhanced production of anti-inflammatory cytokines during re-exposure to malaria [63].

Our observation that children with SMA were younger than those with non-SMA is consistent with previous studies among African children [50, 64]. However, the young age was not observed to affect the levels of the cell populations and cytokines investigated (**see S1 Table**). This observation is also consistent with recent investigations in Malawian children which observed that lymphocyte subset profiles of different forms of severe malaria were independent of age [64]. Furthermore, earlier pediatric studies in an area of high malaria transmission in western Kenya also reported no differences in these cell populations and cytokine production among children aged 0.5 to 5 years [65].

Recent studies showed that reduced protective immunity in C5aR^-/-^ mice was due to impaired function of malaria-specific CD4+ T-cells and consequent differentiation of effector memory (CD62L^low^CD44^high^) and central memory (CD62L^high^CD44^high^) CD4+ T-cells [66]. This underscores the importance of memory CD4+ T-cells in malaria immunity. Moreover, although memory CD4+ T-cells are important in protective immunity against blood-stage parasites, they can also contribute to enhanced pathogenesis [67]. Our observation that children with SMA had decreased levels of memory-like CD4+ T-cells expressing IL-17 suggests that high levels of these cells may be required for protection against SMA pathogenesis or alternatively, they may be sequestered in the deeper tissues where they could result in SMA pathogenesis.

Previous studies in both murine models and humans have reported a strong induction of IFN-γ during acute plasmodial infections [68, 69]. Although IFN-γ can be produced by various cell types, it still remains unclear which cell types are responsible for IFN-γ-induced protection versus pathology [42]. The significantly higher proportion of IFN-γ-producing lymphocytes in children with SMA suggests that elevated lymphocytic production of IFN-γ is associated with enhanced pathogenesis. We have previously shown that children with SMA in this holoendemic region have inefficient erythropoiesis [70]. Since IFN-γ can suppress erythropoiesis [30, 32, 71, 72], enhanced lymphocytic production of IFN-γ in children with SMA may be a central process for enhanced pathogenesis in these children. Our observation that children with SMA also had low RPI may support the role of IFN-γ in suppressing erythropoiesis. However, since low RPI has also been associated with hemolysis in African children with SMA [73], the role of these cytokines in suppressing erythropoiesis needs to be investigated in the context of hemolysis and other anemia causing processes.

Initially, the IL-17/IL-23 pathway was shown to be important in the pathology of inflammation associated with autoimmune diseases [33], and in malaria pathogenesis [36, 38]. Results presented here showing decreased proportions of memory-like CD4+ T-cells expressing IL-17 in children with SMA, and comparable circulating levels of IL-17 in the two groups of children contrasts with previous investigations which, demonstrated that IL-17F levels were elevated with increased malaria severity in pediatric populations of Togo [74]. However, the finding on circulating levels of IL-17 is consistent with other studies in which IL-17 was not found to be associated with cerebral malaria (the phenotype of severe malaria in the region) and mortality in Ghanaian children [75]. Furthermore, our findings also diverge from previous findings which reported association of IL-17 with protection against pregnancy-associated malaria [39] and development of cerebral malaria in *P*. *berghei* infected mice [43]. These disparate findings on the role of IL-17 during malaria infections can be explained by the different pathological mechanisms proposed for the malaria disease outcomes [39, 75, 76] and by Perez-Mazliah and Langhorne’s [42] review report that although Th17 cells are activated during malaria infections, their precise role is yet to be established.

In order to dissect the long term effect of IFN-γ in malaria pathogenesis, we compared the circulating IFN-γ levels between the non-SMA and SMA groups. Our data indicated that children with SMA had higher circulating levels of IFN-γ relative to the non-SMA children from the same population. This observation corroborates the findings of a previous study in western Kenya, in which the SMA phenotype had higher levels of circulating IFN-γ relative to the non-SMA children [53]. Other studies among Thai adults also showed that circulating IFN-γ levels were higher in patients with severe malaria [77]. We therefore, postulate that IFN-γ appears to be protective during early activation and becomes pathologic following chronic parasitemia. This could be due to the fact that as an inflammatory mediator, initial inflammatory responses purpose to control parasite densities. However, it has been reported that depending on the timing and magnitude of inflammatory mediators’ induction, the immunological response to malaria can result to damaging host tissues [76]. Although previous studies reported that IFN-γ levels correlate with control of parasitemia [24], this study did not make such observations.

It has previously been reported that IL-12 promotes differentiation of naïve CD4+ T cells and their subsequent production of IFN-γ while IL-23 induces IL-17 production from activated CD4+ memory T cells [21, 22]. We and others have previously reported that African children with SMA have low levels of circulating IL-12 and increased levels of IL-23 [34–36]. Since IL-12 can suppress IL-23-induced IL-17 production [21, 22] our observation that circulating levels of IFN-γ and IL-17 were negatively correlated suggests that this could possibly be through the IL-12/IL-23 signaling pathway. This could also explain our observation that circulating IFN-γ levels were positively associated with naïve-like CD4+ T cells expressing IFN-γ. Although this model proposes that IL-23 induces activated CD4+ memory T cells to produce IL-17, our observation of low levels of memory-like CD4+ T cells expressing IL-17 in children with SMA may be due to that fact that differentiation of naïve CD4+ T cells to memory CD4+ T cells is accompanied by expression of receptors (CCR7 and/or CD62L) that facilitate their migration and homing to lymphoid organs or inflamed tissues [78, 79]. This pathway could also possibly explain the observed differences in the patterns seen between the naïve-like and memory-like CD4+ T cells and the low circulating IL-17 levels in children with SMA. However, additional studies are still required to confirm the source and role of IL-17 in SMA pathogenesis.

Based on the on-ground facilities in Kenya at the time of the study, we were met with several challenges. For example, we were only capable of examining two cytokines using a single parameter, whereas use of multi-parametric flow cytometry would have allowed for additional insight into the characteristics of CD4+ T-cell or CD8+ T cell populations and their role in malaria-induced pathology e.g., IFN-γ/IL-10 and IFN-γ/IL-17 co-producing cells, etc. In addition, we did not measure the levels of IL-27 that has been shown to suppress IFN-γ production by CD4+ T-cells during malaria infection [80]. Since we used peripheral blood, we do not know the distribution of these cells in different tissues, and their possible impact within the tissues.

In conclusion, we demonstrate that children with SMA have elevated expression of IFN-γ by peripheral lymphocytes. We further demonstrate that children presenting with SMA have decreased levels of memory-like CD4+ T-cells expressing IL-17 and elevated circulating IFN-γ levels. The inverse correlation observed between circulating levels of IFN-γ and IL-17 suggests that both cytokines may be implicated in modulating pathogenesis of SMA. However, future studies are warranted to determine if the responses characterized here are directly or indirectly involved in the suppression of erythropoiesis in children with malarial anemia.

## Supporting information

S1 Fig Representative flow cytometry plots of cytokine production and the gating strategy used to set the cut-offs for cytokine-positive and–negative cells. **(A)** Representative forward versus side scatter gating strategy for the peripheral blood stimulation for the study participants. Dot plot **(B)** CD4+CD45RA+ T cells, **(C)** CD4+CD45RA+IFN-γ+ T cells, **(D)** CD4+CD45RA+IL-17+ T cells, **(E)** CD4+CD45RA-IFN-γ+ T cells, **(F)** CD4+CD45RA-IL-17+ T cells.
(TIF)

S2 Fig Representative flow cytometry plots showing the gating strategy using isotypic controls and matched IFN-γ antibodies to set the cut-offs for cytokine-positive and -negative cells. Isotypic controls 1–3 have their matched IFN-γ-positive cell cut-offs below them.
(TIF)

S3 Fig Representative flow cytometry plots showing the gating strategy using isotypic controls and matched IL-17 antibodies to set the cut-offs for cytokine-positive and -negative cells. Isotypic controls 1–3 have their matched IL-17-positive cell cut-offs below them.
(TIF)

S1 Table Characteristics of the study participants stratified by age. Data analysis performed by Mann-Whitney U tests except for gender and sickle cell trait that we compared using χ^2^ tests. Data are presented as median (IQR); except for gender and sickle cell trait that are presented as n (%).
(DOCX)

## Abbreviations

MFI: mean fluorescence intensity
SMA: severe malarial anemia
non-SMA: non-severe malarial anemia
