# Supplementary figures and images for: CD4 T-cell expression of IFN-γ and IL-17 in pediatric malarial anemia
Source: PLoS One. 2017 Apr 20;12(4):e0175864. doi: 10.1371/journal.pone.0175864 (PMC5398558; doi:10.1371/journal.pone.0175864)

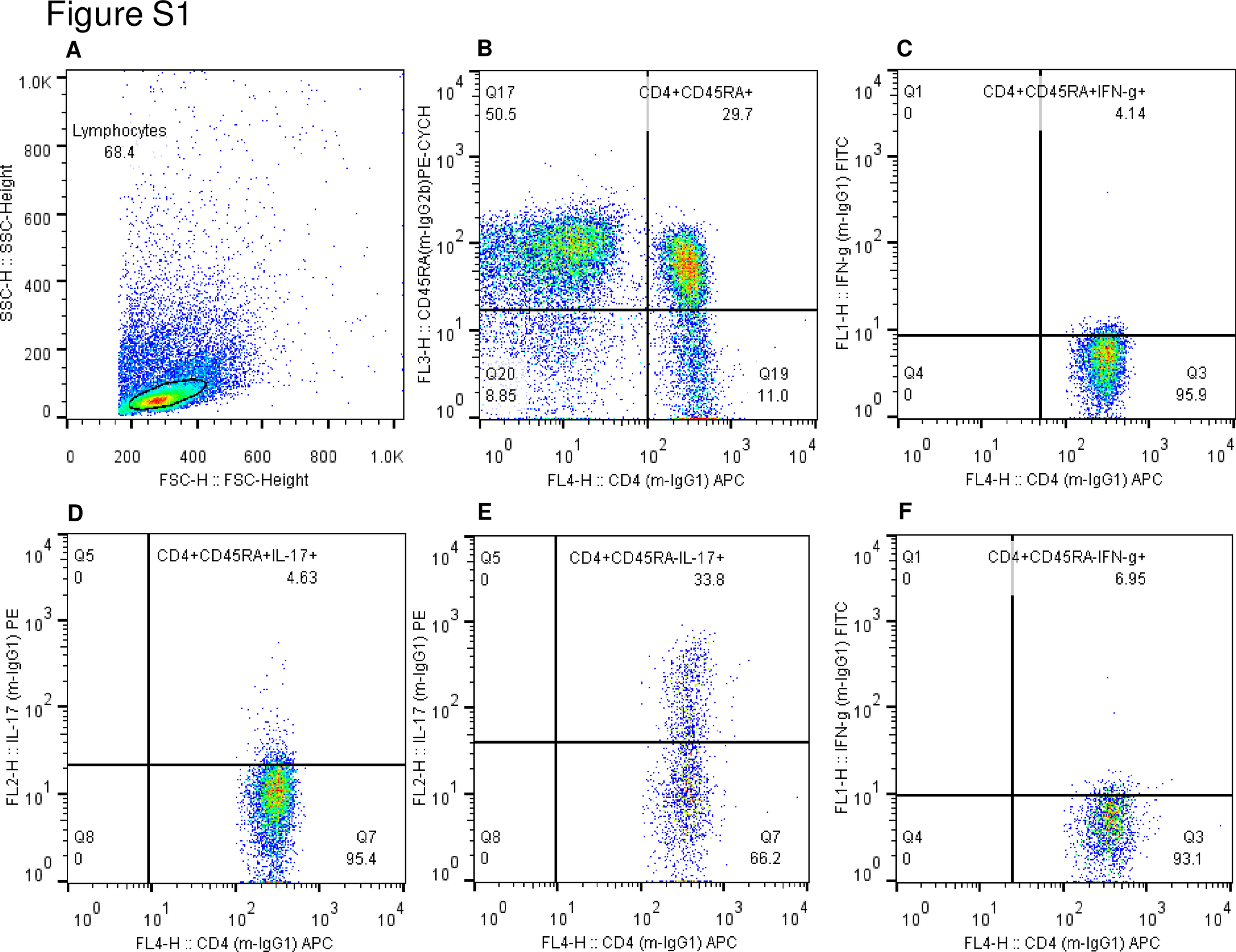

Supplement: S1 Fig — (A) Representative forward versus side scatter gating strategy for the peripheral blood stimulation for the study participants. Dot plot (B) CD4+CD45RA+ T cells, (C) CD4+CD45RA+IFN-γ+ T cells, (D) CD4+CD45RA+IL-17+ T cells, (E) CD4+CD45RA-IFN-γ+ T cells, (F) CD4+CD45RA-IL-17+ T cells. (TIF) [file pone.0175864.s001.tif]

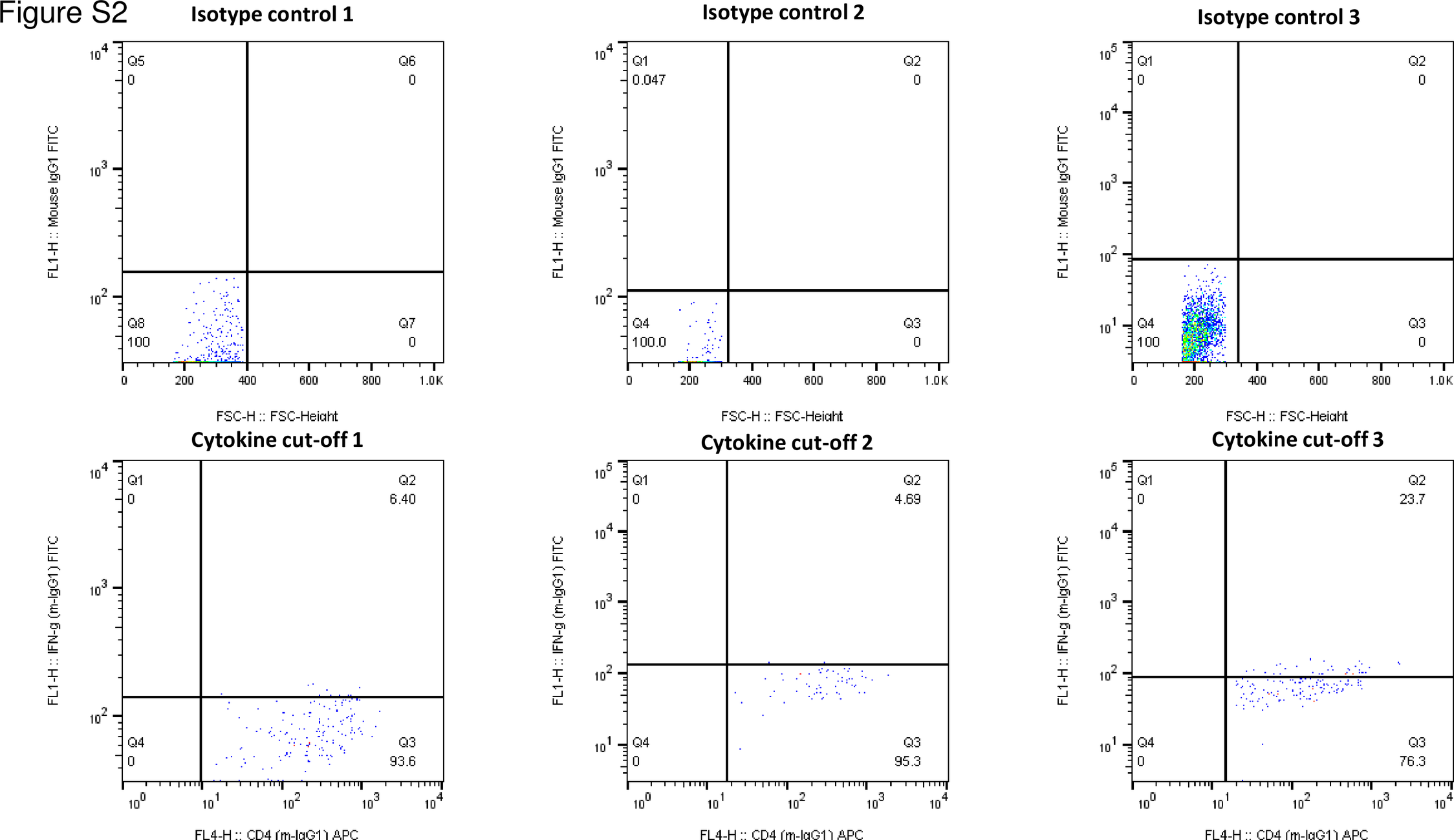

Supplement: S2 Fig — Isotypic controls 1–3 have their matched IFN-γ-positive cell cut-offs below them. (TIF) [file pone.0175864.s002.tif]

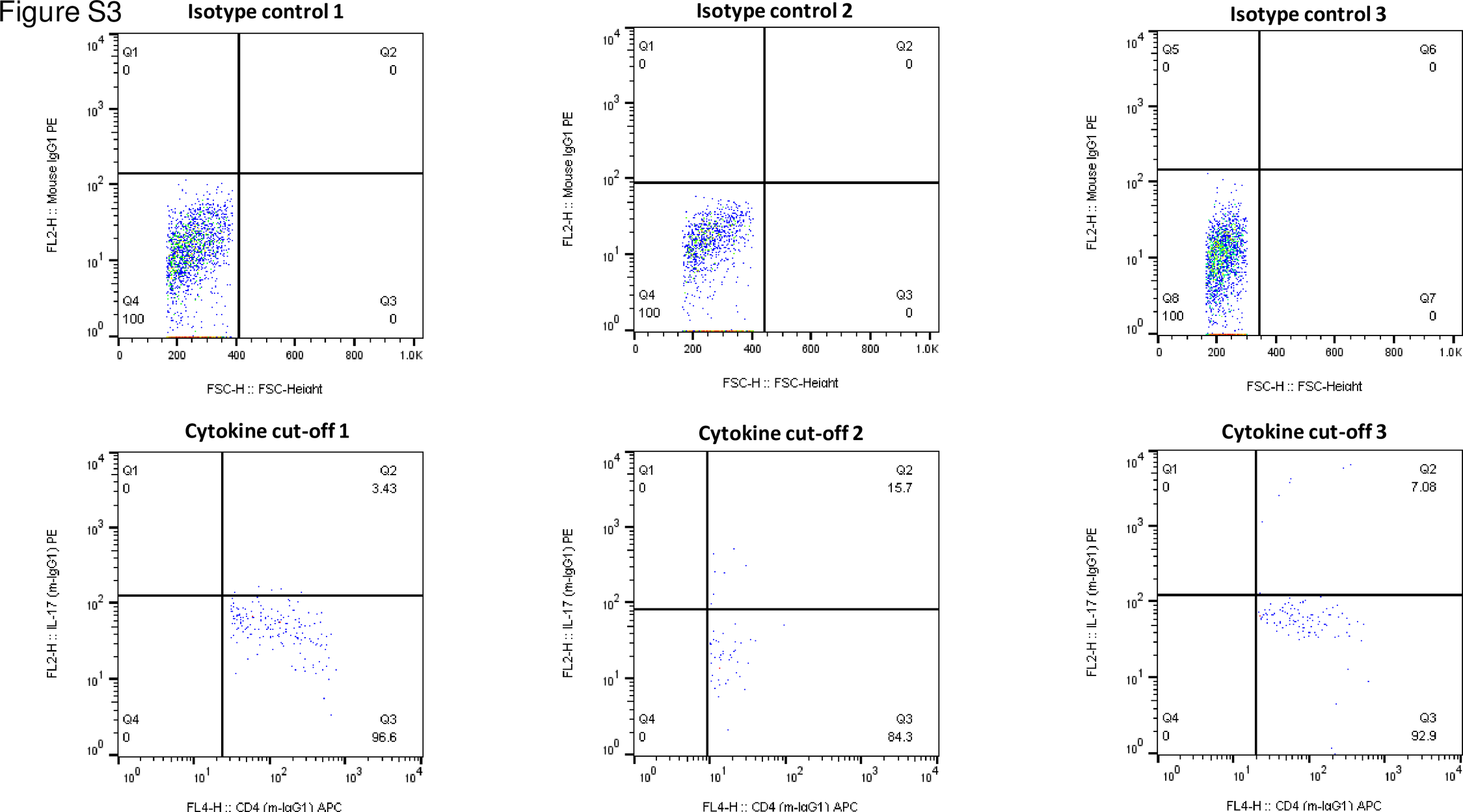

Supplement: S3 Fig — Isotypic controls 1–3 have their matched IL-17-positive cell cut-offs below them. (TIF) [file pone.0175864.s003.tif]
