# Supplementary material for: CD4 T-cell expression of IFN-γ and IL-17 in pediatric malarial anemia
Source: PLoS One. 2017 Apr 20;12(4):e0175864. doi: 10.1371/journal.pone.0175864 (PMC5398558; doi:10.1371/journal.pone.0175864)
Supplement: S1 Table — Data analysis performed by Mann-Whitney U tests except for gender and sickle cell trait that we compared using χ2 tests. Data are presented as median (IQR); except for gender and sickle cell trait that are presented as n (%). (DOCX) [file pone.0175864.s004.docx]

**Table S1: Characteristics of the study participants stratified by age.**

| **Parameter** | **SMA** | | **P value** | **NON-SMA** | | **P value** |
| --- | --- | --- | --- | --- | --- | --- |
|  | Younger (*n*=20) | Older (*n*=19) |  | Younger (*n*=25) | Older (*n*=25) |  |
| Age (months) | 12.5 (5.0) | 26.0 (4.0) | **0.0001** | 17.0 (8.0) | 30.0 (11.0) | **0.0001** |
| Female, n (%) | 6 (35.3) | 11 (64.7) | 0.111 | 10 (38.5) | 15 (66.0) | 0.165 |
| Sickle cell trait, n (%) | 2 (0.1) | 2 (0.1) | 0.999 | 4 (14.4) | 6 (24.0) | 0.499 |
| Temperature, °C | 38.0 (0.7) | 38.1 (1.0) | 0.324 | 38.2 (1.2) | 38.0 (1.9) | 0.468 |
| Hemoglobin (g/dL) | 4.75 (1.8) | 5.00 (1.0) | 0.888 | 9.35 (2.0) | 10.40 (1.8) | 0.065 |
| Parasitemia /µL | 20,962 (106,822) | 36,462 (85,751) | 0.866 | 45,076 (102,680) | 31,000 (210,827) | 0.672 |
| WBC, ×10^9^/L | 14.50 (9.5) | 13.00 (7.0) | 0.876 | 12.00 (6.5) | 9.00 (5.0) | 0.086 |
| Monocyte, ×10^3^/µL | 7.50 (3.0) | 6.00 (5.0) | 0.399 | 7.00 (7.5) | 6.00 (3.5) | 0.777 |
| Granulocytes, ×10^3^/µL | 54.35 (22.3) | 54.55 (8.1) | 0.739 | 54.00(25.8) | 64.10 (23.3) | 0.110 |
| Lymphocytes, ×10^3^/µL | 42.0 (22.0) | 40.0 (8.0) | 0.613 | 39.0 (22.0) | 32.0 (26.0) | 0.572 |
| LymphocytesIFN-γ+ | 8.51 (7.48) | 7.73 (10.01) | 0.628 | 6.17 (4.12) | 6.03 (7.21) | 0.891 |
| LymphocytesIL-17+ | 12.25 (17.33) | 9.85 (13.75) | 0.739 | 7.60 (6.30) | 9.15 (12.55) | 0.670 |
| CD4+CD45RA+IFN-γ+ | 27.90 (35.50) | 25.40 (14.87) | 0.619 | 7.10 (12.30) | 26.70 (28.00) | 0.350 |
| CD4+CD45RA+IL-17+ | 33.50 (25.85) | 26.70 (37.45) | 1.000 | 21.35 (24.38) | 17.10 (22.70) | 0.428 |
| CD4+CD45RA-IFN-γ+ | 1.85 (3.89) | 2.19 (4.31) | 1.000 | 2.08 (4.05) | 3.38 (5.53) | 0.651 |
| CD4+CD45RA-IL-17+ | 3.87 (5.65) | 2.51 (3.20) | 0.104 | 7.31 (14.03) | 4.22 (12.33) | 0.072 |
| Circulating IFN-γ levels | 22.22 (0.0) | 22.21 (0.0) | 0.335 | 10.25 (4.84) | 10.97 (14.57) | 0.176 |
| Circulating IL-17 levels | 35.12 (54.8) | 14.46 (30.4) | 0.335 | 28.25 (112.3) | 14.77 (15.5) | 1.000 |

Data analysis performed by Mann-Whitney U tests except for gender and sickle cell trait that we compared using χ^2^ tests. Data are presented as median (IQR); except for gender and sickle cell trait that are presented as n (%).
